# Dietary Antioxidant Quality Score and Epilepsy Odds in the US Adults: A Cross‐Sectional NHANES Study

**DOI:** 10.1002/brb3.71018

**Published:** 2025-10-29

**Authors:** Hamid Abbasi, Sara Khoshdooz, Mohammad Mehdi Abbasi, Ghazaleh Eslamian

**Affiliations:** ^1^ Faculty of Medicine Guilan University of Medical Science Rasht Iran; ^2^ Student Research Committee, Faculty of Nutrition and Food Technology Shahid Beheshti University of Medical Sciences Tehran Iran; ^3^ Department of Cellular and Molecular Nutrition, Faculty of Nutrition and Food Technology, National Nutrition and Food Technology Research Institute Shahid Beheshti University of Medical Sciences Tehran Iran

**Keywords:** antioxidant, dietary antioxidant quality score, epilepsy, NHANES, oxidative stress

## Abstract

**Background:**

Epilepsy is defined by the occurrence of a minimum of two unprovoked seizures, with a temporal gap exceeding 24 h between episodes. This research aimed to ascertain the link between dietary antioxidant quality score (DAQS) and the odds of developing epilepsy.

**Methods:**

In this cross‐sectional study, utilizing data from the National Health and Nutrition Examination Survey spanning 2017–2020, binary logistic regression analyses were carried out to investigate the link between DAQS and the odds of epilepsy. We recruited a cohort of 1086 individuals with pathologically confirmed diagnoses of epilepsy with 1086 controls.

**Results:**

The mean age of participants was 58.26 years, with cases and controls averaging 54.26 and 62.26 years, respectively. Highest adherence to the DAQS was significantly linked to epilepsy compared to their counterparts with the lowest adherence (odds ratio [OR]: 0.76, 95% confidence interval [CI]: 0.68–0.85, *p* < 0.001), subgroup results revealed higher odds in those refusing education (OR: 5.6, 95% CI: 1.00–10.6, *p* = 0.05) and never married individuals (OR: 1.63, 95% CI: 1.22–2.18, *p* = 0.001). Conversely, significant reductions were observed for education levels beyond nineth grade (e.g., OR: 0.33 for high school graduates, *p* <0.001) and widowed participants (OR: 0.71, 95% CI: 0.56–0.91, *p* = 0.006). Ethnically, non‐Hispanic Blacks and Asians had 36% (OR: 0.64, 95% CI: 0.44–0.94, *p* = 0.024) and 65% (OR: 0.35, 95% CI: 0.20–0.60, *p*<0.001) lower odds than Mexican Americans.

**Conclusion:**

Our findings suggest a modest inverse association between higher DAQS and epilepsy odds; however, due to the cross‐sectional design, no causal inference can be made. Sociodemographic factors appear to modify this association and should be considered in future research.

## Graphical abstract

This cross‐sectional study establishes a significant inverse link between dietary antioxidant quality score (DAQS) and epilepsy risk using NHANES 2017–2020 data, revealing a 24% reduced odds of epilepsy with high DAQS adherence and demographic variations in protective effects.

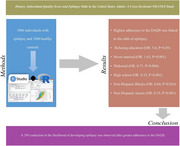

**Abbreviations**
ANCOVA: analysis of covariance
ANOVA: analysis of variance
BMI: body mass index
CDAI: composite dietary antioxidant index
CDC: Center for Disease Control and Prevention
CI: confidence interval
DAQS: dietary antioxidant quality score
FNDDS: Food and Nutrient Databases for Dietary Studies
GSH: glutathione
HDL: high‐density lipoprotein
ICD: International Classification of Diseases
KD: ketogenic diet
LDL: low‐density lipoprotein
MEC: mobile examination center
NHANES: National Health and Nutrition Examination Survey
OR: odds ratio
RDI: recommended dietary intakes
RNS: reactive nitrogen species
ROS: reactive oxygen species
SOD: superoxide dismutase
TIBC: total iron binding capacity

## 1 Introduction

Epilepsy is a common neurological disorder marked by recurrent seizures, which can affect cognitive, physical, and emotional well‐being (Christensen et al. 2023; Sultana et al. 2021). Identifying modifiable risk factors is essential for improving outcomes and guiding public health strategies (Beghi 2020). Understanding its etiology and developing effective prevention and treatment strategies are crucial, given its global burden (Kobau et al. 2024; Moura et al. 2022).

While epilepsy's causes are multifactorial, identifying modifiable risk factors is crucial. Emerging evidence highlights the potential role of dietary components in managing and preventing epilepsy. In particular, antioxidants have garnered attention for their potential to combat oxidative stress, a key factor in neuronal damage and seizure activity (Falco‐Walter 2020; Balestrini et al. 2021, E. Perucca et al. 2023, P. Perucca et al. 2020). Oxidative stress results from an imbalance between free radicals and antioxidants, leading to cellular damage that may contribute to epilepsy pathogenesis (Valko et al. 2007).

Antioxidants found in fruits, vegetables, and whole grains play a critical role in maintaining cellular health and mitigating oxidative damage. Past studies have explored the link between epilepsy and specific antioxidant‐rich foods or micronutrients, suggesting that a diet high in antioxidants may reduce seizure frequency and improve neurological outcomes (Kim and Cho 2019, Francis and Stevenson 2018, Y. Zhang et al. 2024; Sondhi et al. 2020, X. Yang et al. 2024; Jang et al. 2016; Madireddy and Madireddy 2023). The growing evidence underscores the cumulative and synergistic impact of dietary antioxidants, emphasizing the need for comprehensive assessments of total antioxidant intake rather than individual nutrient studies (Martinc et al. 2014; Parsons et al. 2022; Lin et al. 2020; Alonso‐Castro et al. 2019; Nassiri‐Asl et al. 2013; El‐Rashidy et al. 2020).

Yet, research on the overall impact of dietary antioxidants on epilepsy is limited. A recent cross‐sectional study revealed higher levels of a composite dietary antioxidant index were associated with reduced epilepsy risk (Y. Zhang et al. 2024). Additionally, X. Yang et al. 2024 found individuals with epilepsy had lower dietary oxidative balance scores compared to the general population, highlighting the link between diet and epilepsy risk.

The dietary antioxidant quality score (DAQS) is a novel tool assessing the cumulative impact of specific dietary antioxidants on health outcomes by assigning scores based on adherence to FDA's recommended daily intake values (Rivas et al. 2012; Shi and Fang 2024). This scoring system offers a practical approach to evaluating dietary patterns concerning antioxidant consumption.

While other indices such as the composite dietary antioxidant index (CDAI) have been employed in prior studies, the DAQS offers distinct advantages by focusing on adherence to recommended daily intakes of essential antioxidants, facilitating public health interpretation and clinical application (Rivas et al. 2012; Shi and Fang 2024). Unlike CDAI, which involves a *z*‐score‐based standardization sensitive to population‐specific distributions, the DAQS uses absolute intake thresholds, making it more practical for dietary counseling (Y. Chen et al. 2023; Wang and Yi 2022).

The biological plausibility of dietary antioxidants influencing epilepsy is supported by extensive evidence (Chidambaram et al. 2024, N. Yang et al. 2020; Parsons et al. 2022; Madireddy and Madireddy 2023). Oxidative stress has been implicated in neuronal hyperexcitability and seizure pathophysiology through mechanisms involving mitochondrial dysfunction, glutamate excitotoxicity, and impaired antioxidant defense systems (Parsons et al. 2022). Antioxidants such as vitamin E, glutamine, and selenium play critical neuroprotective roles by neutralizing reactive oxygen species (ROS) and modulating inflammatory cascades, which are commonly elevated in epileptic conditions (Chidambaram et al. 2024, N. Yang et al. 2020; Madireddy and Madireddy 2023). Specific antioxidant pathways such as glutathione (GSH), which detoxifies hydrogen peroxide via glutathione peroxidase, and superoxide dismutase (SOD), which converts superoxide radicals into less reactive molecules, are crucial in mitigating oxidative neuronal injury during seizures (Liang et al. 2024, X. Zhang et al. 2022).

To date, no study has comprehensively examined the relationship between DAQS and epilepsy risk. Our study, using National Health and Nutrition Examination Survey (NHANES) 2017–2020 databases, aims to address this gap by exploring the potential protective role of DAQS in epilepsy risk and shedding light on dietary choices' broader implications for individuals managing or predisposed to epilepsy.

## 2 Methods

### 2.1 Study Design and Participants

**FIGURE 1:**
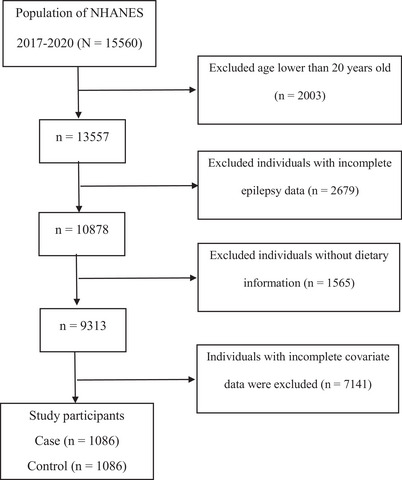
Flow diagram of the cross‐sectional study using NHANES 2017–2020.

The NHANES is a comprehensive series of investigations designed to assess the health and nutritional profiles of the US population. This study utilized data from the 2017–2018 and 2019–2020 cycles of NHANES to achieve a sufficient sample size. Participants, who lacked dietary information or epilepsy status, as well as those with incomplete datasets regarding potential confounders, were systematically excluded. Confounding factors include gender; age; ethnicity; educational attainment; marital status; body mass index (BMI); energy intake; waist circumference; hip circumference; high‐density lipoprotein (HDL); low‐density lipoprotein (LDL); triglycerides; total cholesterol; and ferritin, iron, and total iron binding capacity (TIBC) serum concentrations. The study employed a cross‐sectional design with a sample size of 2172 participants (Figure 1). NHANES, initiated by the Center for Disease Control and Prevention (CDC) in 1971, uses a sophisticated probability sampling methodology to ensure representation of noninstitutionalized community‐dwelling residents across the United States. The de‐identified nature of the data exempted this research from institutional review board oversight. Detailed survey protocols and procedural manuals are available at https://www.cdc.gov/nchs/nhanes/index.htm.

### 2.2 Dietary Nutrient Data

The USDA's Food and Nutrient Databases for Dietary Studies (FNDDS) spanning 2007–2014 were utilized for analyzing dietary intakes during this period (http://www.ars.usda.gov/ba/bhnrc/fsrg). The FNDDS contains a vast amount of data that help code individual food items, portion sizes, and nutrients for calculating dietary intakes. Since the FNDDS is used to generate nutrient intake data for the NHANES “What We Eat in America” component, there is no need for additional estimations. Researchers can access the FNDDS to examine the nutrient content of specific foods and beverages, including portion sizes and preparation methods, allowing for detailed analysis.

The dietary intake data included total nutrient intakes from two dietary interviews: the dietary interview—total nutrient intakes for both the first and second days and dietary supplement intakes from the dietary supplement use 30‐day‐total dietary supplements dataset. Total nutrient intakes were assessed using a 24‐h dietary recall method, capturing food consumption over the previous day. Initial interviews took place in the NHANES mobile examination center (MEC), with follow‐up interviews occurring 3–10 days later to avoid overlap with MEC interviews. Average intake was calculated for participants who completed two 24‐h recalls, while a single reliable recall was used for others.

### 2.3 DAQS Calculation

The DAQS was developed from a selection of vitamins and minerals recognized for their antioxidant properties, including selenium, zinc, vitamin A, vitamin C, and vitamin E.(Tur et al. 2005) In creating the DAQS, we compared daily nutrient intakes to established recommended dietary intakes (RDI) (https://health.gov/sites/default/files/2019‐09/2015‐2020_Dietary_Guidelines.pdf). Each of the five antioxidants was assessed individually using a binary scoring system (0 or 1) for each component. Following the methodology proposed by Tur et al. (2005), an intake level below two‐thirds of the RDI received a score of 0, while an intake exceeding two‐thirds of the RDI was given a score of 1. As a result, the total DAQS could range from 0 (indicating very poor dietary quality) to 5 (representing high dietary quality) (Aranceta et al. 2001).

### 2.4 Definition of Epilepsy

Epilepsy‐related data were extracted from NHANES 2017–2020 through the designated section on “Prescription Drugs.” Participants were asked about their use of any medications in the past 30 days specifically for the management of “epilepsy and recurrent seizures,” as identified by the International Classification of Diseases (ICD) code G40. For this study, individuals who reported using these medications were categorized as having epilepsy (Ran et al. 2024). We recognize the limitation that our operational definition of epilepsy, based on prescription drugs, may lead to misclassification by both excluding undiagnosed cases and including individuals with non‐epileptic events. This may result in measurement bias and influence the observed associations.

### 2.5 Covariates

In this cross‐sectional study, various covariates potentially influencing dietary nutrient intake and epilepsy prevalence were systematically gathered. The covariates encompassed demographic variables such as age (in years), gender (male or female), and racial/ethnic classification (Mexican American, Other Hispanic, Non‐Hispanic White, Non‐Hispanic Black, Other Races). Additionally, marital status was categorized into several groups: married, widowed, divorced, separated, never married, and cohabiting with a partner. Educational attainment was stratified into distinct levels: less than nineth grade, 9th–11th grade, high school diploma, some college or associate degree, and college graduate or higher. Other relevant health metrics included BMI (in kg/m^2^); waist circumference (cm); hip circumference (cm); HDL (in mg/dL); LDL (in mg/dL); triglycerides (mg/dL); total cholesterol (mg/dL); serum concentrations of ferritin (ng/dL), iron (µg/dL), and TIBC (in µg/dL); and dietary intake (in grams per day). Furthermore, comprehensive assessments of dietary macronutrient and micronutrient consumption (in grams per day) and overall energy intake (in kilocalories per day) were conducted.

### 2.6 Statistical Analysis

Initially, we established quantile cutoff thresholds for the DAQS within the control group. Subsequently, we stratified all study participants based on these defined cutoffs. The quantiles of the DAQS were delineated as follows: Q1 < 0.004, Q2 (0.004–0.04), Q3 (0.04–0.17), Q4 (0.17–0.47), and Q5 > 0.47. To assess differences in continuous variables, independent samples student's *t*‐tests were employed, while chi‐square tests were used to examine the distribution of categorical variables between the epileptic case and control groups. Comparisons across quantiles of the DAQS were performed using one‐way analysis of variance (ANOVA) and chi‐square tests, as appropriate. Age and sex‐adjusted nutrient intakes across DAQS quantiles were calculated using analysis for covariance (ANCOVA). Binary logistic regression analyses were carried out to investigate the link between DAQS and the odds of epilepsy. Four distinct regression models were utilized: the first model was unadjusted and considered the crude model, the second model adjusted for age (as a continuous variable) and gender (male/female), the third model further adjusted for education level, and the final model included additional adjustments for race and marital status. The first quantile of DAQS was designated as the reference category, from which odds ratios (ORs) and 95% confidence intervals (CIs) for the other quantiles were derived. The overall trend in ORs across DAQS quantiles was assessed by defining quantiles of DAQS as ordinal variables. Statistical analyses were executed using RStudio version 2024.09.0 in conjunction with R version 4.3.3. Relevant R packages, including “glm,” “t.test,” “chisq.test,” and “anova,” were employed for the analyses. A *p*‐value of ≤ 0.05 was considered statistically significant.

## 3 Results

### 3.1 Baseline Characteristics

In total, our cross‐sectional study included 2172 individuals. The average age of participants was 58.26 years. Table 1 summarizes the general characteristics of epileptic patients and controls. Table 2 presents selected antioxidant‐related biochemical markers (HDL‐c, ferritin, iron, and TIBC) across DAQS quantiles and epilepsy status. Additional biochemical and anthropometric characteristics, including BMI, LDL‐c, triglycerides, and total cholesterol, are provided in Table S1. Significant differences were found in age; race; marital status; education level; BMI; waist circumference; hip circumference; HDL; triglycerides; total cholesterol; and serum concentrations of ferritin, iron, and TIBC (*p* < 0.05) between participants with and without epilepsy. Individuals with epilepsy were more likely to have less than a nineth grade education (*p* < 0.001), be of Mexican American race (*p *< 0.05), never married (*p* < 0.001), have a lower BMI (28.85 ± 6.52 vs. 31.46 ± 7.84, *p *< 0.001), waist circumference (100.39 ± 18.17 vs. 105.98 ± 16.85, *p *< 0.001), hip circumference (104.99 ± 14.06 vs. 110.21 ± 15.19, *p* < 0.001), higher HDL levels (61.39 ± 24.28 vs. 53.11 ± 16.46, *p *< 0.001), lower triglycerides (104.88 ± 58.53 vs. 125.65 ± 100.58, *p *< 0.001), lower ferritin concentrations (111.34 ± 93.47 vs. 164.33 ± 208.29, *p *< 0.001), and lower TIBC levels (315.27 ± 62.09 vs. 322.67 ± 53.75, *p *< 0.05).

**TABLE 1:** General characteristics of cases and controls stratified by quantiles of the dietary antioxidant quality score.

Characteristic	Epilepsy		Energy‐adjusted dietary antioxidant quality score					*p*‐value^b^
	Yes *n* = 1086^a^	No *n* = 1086^a^	Q1 *n* = 424^a^	Q2 *n* = 416^a^	Q3 *n* = 370^a^	Q4 *n* = 423^a^	Q5 *n* = 461^a^	
Age (year)	54.26 ± 17.99	62.26 ± 13.55	58.49 ± 17.38	57.59 ± 16.82	57.98 ± 15.92	58.65 ± 15.49	58.71 ± 16.34	< 0.0001
Gender								0.828
Female	614 (56.54)	609 (56.08)	264 (61.97)	238 (57.21)	214 (57.84)	240 (56.74)	231 (50.11)	
Male	472 (43.46)	477 (43.92)	162 (38.03)	178 (42.79)	156 (42.16)	183 (43.26)	230 (49.89)	
Education								< 0.0001
9th–11th grade (includes 12th grade with no diploma)	192 (18.06)	120 (11.10)	60 (14.39)	67 (16.38)	59 (16.03)	61 (14.56)	59 (12.94)	
College graduate or above	205 (19.29)	242 (22.39)	100 (23.98)	66 (16.14)	88 (23.91)	96 (22.91)	93 (20.39)	
Don't Know	13 (1.22)	1 (0.09)	4 (0.96)	2 (0.49)	2 (0.54)	1 (0.24)	4 (0.88)	
High school graduate/general education development or equivalent	277 (26.06)	255 (23.59)	89 (21.34)	106 (25.92)	98 (26.63)	106 (25.30)	103 (22.59)	
Less than nineth grade	137 (12.89)	69 (6.38)	40 (9.59)	37 (9.05)	23 (6.25)	39 (9.31)	49 (10.75)	
Some college or associate of arts degree	239 (22.48)	394 (36.45)	124 (29.74)	131 (32.03)	98 (26.63)	116 (27.68)	148 (32.46)	
Race								< 0.05
Mexican American	141 (12.98)	91 (8.38)	50 (11.74)	45 (10.82)	28 (7.57)	45 (10.64)	60 (13.02)	
Non‐Hispanic Asian	45 (4.14)	69 (6.35)	24 (5.63)	16 (3.85)	17 (4.59)	27 (6.38)	19 (4.12)	
Non‐Hispanic Black	275 (25.32)	278 (25.60)	104 (24.41)	96 (23.08)	98 (26.49)	111 (26.24)	115 (24.95)	
Non‐Hispanic White	453 (41.71)	507 (46.69)	177 (41.55)	206 (49.52)	171 (46.22)	182 (43.03)	199 (43.17)	
Other Hispanic	119 (10.96)	93 (8.56)	55 (12.91)	38 (9.13)	40 (10.81)	36 (8.51)	38 (8.24)	
Other race—including multiracial	53 (4.88)	48 (4.42)	16 (3.76)	15 (3.61)	16 (4.32)	22 (5.20)	30 (6.51)	
Marital status								< 0.0001
Married/living with partner	531 (49.95)	604 (55.87)	221 (53.00)	216 (52.81)	213 (57.88)	226 (53.94)	227 (49.78)	
Never married	282 (26.53)	114 (10.55)	80 (19.18)	70 (17.11)	67 (18.21)	71 (16.95)	85 (18.64)	
Widowed/divorced/separated	250 (23.52)	363 (33.58)	116 (27.82)	123 (30.07)	88 (23.91)	122 (29.12)	144 (31.58)	

*Note*: Data are presented as *n* (%) or mean ± SD.

^a^ Individuals DAQS score: first quantile: less than 0.004; second quantile: between 0.004 and 0.04; third quantile: between 0.04 and 0.17; fourth quantile: between 0.17 and 0.47; and fifth quantile: more than 0.47.

^b^ Derived from independent Student's *t*‐test or Pearson's *χ* ^2^ test, as applicable.

**TABLE 2:** Antioxidant‐related biochemical markers among epilepsy cases and controls across DAQS quantiles.

	Epilepsy		Energy‐adjusted dietary antioxidant quality score					*p*‐value^b^
	Yes *n* = 1086^a^	No *n* = 1086^a^	Q1 *n* = 424^a^	Q2 *n* = 416^a^	Q3 *n* = 370^a^	Q4 *n* = 423^a^	Q5 *n* = 461^a^	
HDL‐c (mg/dL)	61.39 ± 24.28	53.11 ± 16.46	55.22 ± 18.57	59.28 ± 23.02	58.58 ± 22.40	56.71 ± 19.94	56.45 ± 21.26	< 0.0001
Ferritin (ng/dL)	111.34 ± 93.47	164.33 ± 208.29	148.85 ± 161.46	139.34 ± 173.39	124.81 ± 122.52	137.59 ± 197.83	137.77 ± 151.54	< 0.0001
Iron (µg/dL)	84.99 ± 32.81	81.09 ± 31.64	84.24 ± 33.48	86.22 ± 32.64	82.05 ± 30.63	82.73 ± 32.53	80.86 ± 31.71	< 0.0001
TIBC (µg/dL)	315.27 ± 62.09	322.67 ± 53.75	319.87 ± 60.11	317.67 ± 56.14	316.48 ± 54.89	320.07 ± 56.30	319.92 ± 62.69	< 0.05

*Note*: Data are presented as mean ± SD.

Abbreviations: HDL‐c, high‐density lipoprotein‐cholesterol; TIBC, total iron‐binding capacity.

^a^ Individuals DAQS score: first quantile: less than 0.004; second quantile: between 0.004 and 0.04; third quantile: between 0.04 and 0.17; fourth quantile: between 0.17 and 0.47; and fifth quantile: more than 0.47.

^b^ Derived from independent Student's *t*‐test.

The dietary nutrient intakes of participants with and without epilepsy, presented in Table 3, showed that individuals with epilepsy had higher intakes of carbohydrate, vitamin A, thiamine, riboflavin, niacin, vitamin B6, folic acid, vitamin B12, calcium, phosphorus, magnesium, copper, potassium, and energy from diets (*p *< 0.05), while consuming lower amounts of zinc and selenium compared to controls (*p* < 0.05).

**TABLE 3:** Dietary nutrient intake of study population stratified by quantiles of the dietary antioxidant quality score.

	Epilepsy		Energy‐adjusted dietary antioxidant quality score					*p*‐value^b^
	Yes *n* = 1086^a^	No *n* = 1086^a^	Q1 *n* = 426^a^	Q2 *n* = 416^a^	Q3 *n* = 370^a^	Q4 *n* = 423^a^	Q5 *n* = 461^a^	
Energy (kcal/d)	2097.49 ± 981.48	1987.52 ± 882.81	1908.26 ± 814.42	2028.09 ± 818.34	1974.79 ± 842.80	2068.20 ± 946.06	2206.74 ± 1148.09	0.007
Macronutrients								
Protein (g)	75.18 ± 34.82	77.38 ± 37.68	72.17 ± 31.43	73.27 ± 30.79	71.48 ± 33.07	77.03 ± 35.63	86.05 ± 45.32	0.165
Carbohydrate (g)	250.12 ± 107.36	228.43 ± 109.71	225.91 ± 100.49	239.62 ± 102.88	233.16 ± 99.43	239.81 ± 105.66	255.03 ± 129.20	< 0.001
Total fat (g)	80.44 ± 41.01	83.53 ± 45.14	76.91 ± 40.64	81.94 ± 41.56	80.74 ± 41.85	84.11 ± 45.68	85.86 ± 45.16	0.101
Micronutrients								
Vitamin E (mg)	7.38 ± 3.55	9.18 ± 6.74	7.65 ± 4.43	8.41 ± 6.53	8.02 ± 5.00	8.41 ± 5.61	8.90 ± 5.51	< 0.001
Vitamin A (µg)	925.92 ± 1789.83	709.71 ± 900.44	721.92 ± 1101.14	806.48 ± 1604.10	751.78 ± 1270.84	823.89 ± 1370.41	957.05 ± 1618.76	< 0.001
Thiamin (mg)	1.68 ± 0.88	1.50 ± 0.74	1.51 ± 0.72	1.53 ± 0.74	1.47 ± 0.69	1.63 ± 0.83	1.78 ± 1.00	< 0.001
Riboflavin (mg)	2.42 ± 1.94	1.94 ± 1.09	1.98 ± 1.26	2.10 ± 1.42	1.92 ± 1.28	2.18 ± 1.51	2.62 ± 2.12	< 0.001
Niacin (mg)	25.53 ± 16.42	23.78 ± 13.02	22.97 ± 13.47	24.34 ± 15.12	22.62 ± 12.71	24.48 ± 13.80	28.24 ± 17.40	0.007
Vitamin B6 (mg)	2.18 ± 1.59	1.95 ± 1.30	1.88 ± 1.31	2.07 ± 1.54	1.86 ± 1.15	2.02 ± 1.28	2.42 ± 1.78	0.003
Folic acid (µg)	157.38 ± 171.16	138.69 ± 125.76	148.61 ± 153.00	148.37 ± 163.53	125.86 ± 126.71	144.46 ± 133.76	167.66 ± 163.69	< 0.001
Vitamin B12 (µg)	6.64 ± 18.89	4.70 ± 8.36	4.59 ± 10.44	6.23 ± 17.22	4.80 ± 13.18	5.36 ± 14.00	7.07 ± 16.59	0.002
Vitamin C (mg)	82.99 ± 60.53	83.30 ± 78.49	78.41 ± 67.24	77.37 ± 70.96	82.35 ± 64.65	84.31 ± 73.43	92.30 ± 72.72	0.919
Vitamin D (µg)	4.96 ± 7.63	4.65 ± 5.17	4.18 ± 3.91	4.32 ± 5.77	3.91 ± 4.40	5.09 ± 6.44	6.26 ± 9.56	0.286
Vitamin K (µg)	140.97 ± 209.87	140.57 ± 179.26	135.09 ± 203.78	123.02 ± 158.96	153.39 ± 208.38	152.20 ± 202.73	141.41 ± 196.81	0.962
Calcium (mg)	1012.42 ± 953.70	880.14 ± 505.08	877.08 ± 501.60	859.44 ± 487.87	845.03 ± 554.22	952.08 ± 759.82	1160.21 ± 1166.54	< 0.001
Phosphorus (mg)	1377.81 ± 882.79	1296.31 ± 606.32	1244.26 ± 548.71	1266.20 ± 556.73	1245.63 ± 616.70	1356.90 ± 742.44	1539.29 ± 1084.22	0.013
Magnesium (mg)	305.72 ± 180.51	291.68 ± 136.76	279.57 ± 136.37	283.93 ± 129.93	289.09 ± 147.54	308.14 ± 163.58	328.32 ± 201.27	0.044
Iron (mg)	14.23 ± 7.65	13.65 ± 7.46	13.76 ± 7.94	13.59 ± 7.28	13.14 ± 7.39	13.86 ± 7.02	15.13 ± 7.94	0.077
Zinc (mg)	9.80 ± 4.74	10.42 ± 7.15	9.49 ± 5.30	9.94 ± 6.89	9.38 ± 4.85	10.03 ± 5.38	11.49 ± 7.21	0.020
Copper (mg)	1.57 ± 3.32	1.23 ± 1.41	1.20 ± 1.80	1.46 ± 3.02	1.35 ± 2.31	1.42 ± 2.46	1.53 ± 2.88	0.002
Sodium (mg)	3162.82 ± 1298.01	3167.03 ± 1530.39	3077.20 ± 1352.99	3099.86 ± 1446.21	2972.66 ± 1309.92	3234.88 ± 1410.20	3394.96 ± 1519.82	0.945
Potassium (mg)	2915.63 ± 1710.43	2608.64 ± 1200.30	2542.15 ± 1174.93	2638.79 ± 1136.45	2664.79 ± 1277.53	2832.77 ± 1483.70	3080.04 ± 2013.85	< 0.001
Selenium (µg)	102.24 ± 44.66	108.07 ± 56.59	101.26 ± 44.81	100.39 ± 48.62	98.65 ± 49.20	106.10 ± 52.75	117.59 ± 56.56	0.009

*Note*: Data are presented as mean ± SD.

^a^ Individuals DAQS score: first quantile: less than 0.004; second quantile: between 0.004 and 0.04; third quantile: between 0.04 and 0.17; fourth quantile: between 0.17 and 0.47, and fifth quantile: more than 0.47.

^b^ All values were statistically adjusted for gender, age, and energy, with the exception of energy intake, which was adjusted solely for gender and age through the application of analysis of covariance (ANCOVA).

### 3.2 Association Between the DAQS and the Odds of Epilepsy

Four distinct levels of adjustment were employed to evaluate the odds of epilepsy across various quantiles of the DAQS, as illustrated in Table 4. In the unadjusted model, participants in the highest quantile of DAQS exhibited odds of epilepsy that were not significantly lower than those in the lowest quantile (OR: 0.83, 95% CI: 0.64–1.09, *p* = 0.188). Subsequent adjustment for potential confounders, including age, gender, educational level, race, and marital status revealed that individuals demonstrating the highest adherence to the DAQS were significantly linked to epilepsy compared to their counterparts with the lowest adherence (OR: 0.76, 95% CI: 0.68–0.85, *p *< 0.001).

**TABLE 4:** Odds ratios (ORs) and 95% confidence intervals (95% CIs) for the odds of epilepsy stratified by quantiles of energy‐adjusted dietary antioxidant quality scores.

DAQS	Energy‐adjusted dietary antioxidant quality scores						
	Model 1 OR (95% CI)	*p*‐value	Model 2 OR (95% CI)	*p*‐value	Model 3 OR (95% CI)	*p*‐value	Model 4 OR (95% CI)	*p*‐value
Q1	Reference		Reference		Reference		Reference	
Q2	1.01 (0.78, 1.31)	0.944	1.01 (0.77,1.33)	0.950	1.00 (0.75,1.32)	0.867	0.99 (0.75,1.32)	0.954
Q3	0.86 (0.66, 1.13)	0.287	0.86 (0.65, 1.13)	0.272	0.85 (0.64, 1.13)	0.322	0.86 (0.64, 1.15)	0.300
Q4	0.93 (0.71, 1.23)	0.629	0.91 (0.68, 1.21)	0.526	0.91 (0.67, 1.22)	0.558	0.90 (0.66, 1.21)	0.476
Q5	0.83 (0.64, 1.09)	0.188	0.80 (0.60, 1.06)	0.114	0.78 (0.58, 1.04)	0.147	0.76 (0.68, 0.85)	**< 0.001**

*Note*: Binary logistic regression analysis was employed to derive ORs and their corresponding 95% CIs. The overarching trend of the OR across ascending quantiles was investigated by regarding the median score within each quantile as a continuous predictor variable. Model 1: crude model, Model 2: adjusted for gender and age, Model 3: additional adjustments were applied for education level, and Model 4: additional adjustments were applied for race and marital status.

Abbreviations: CI, confidence interval; OR, odds ratio.

### 3.3 Subgroup and Sensitivity Analyses

**FIGURE 2:**
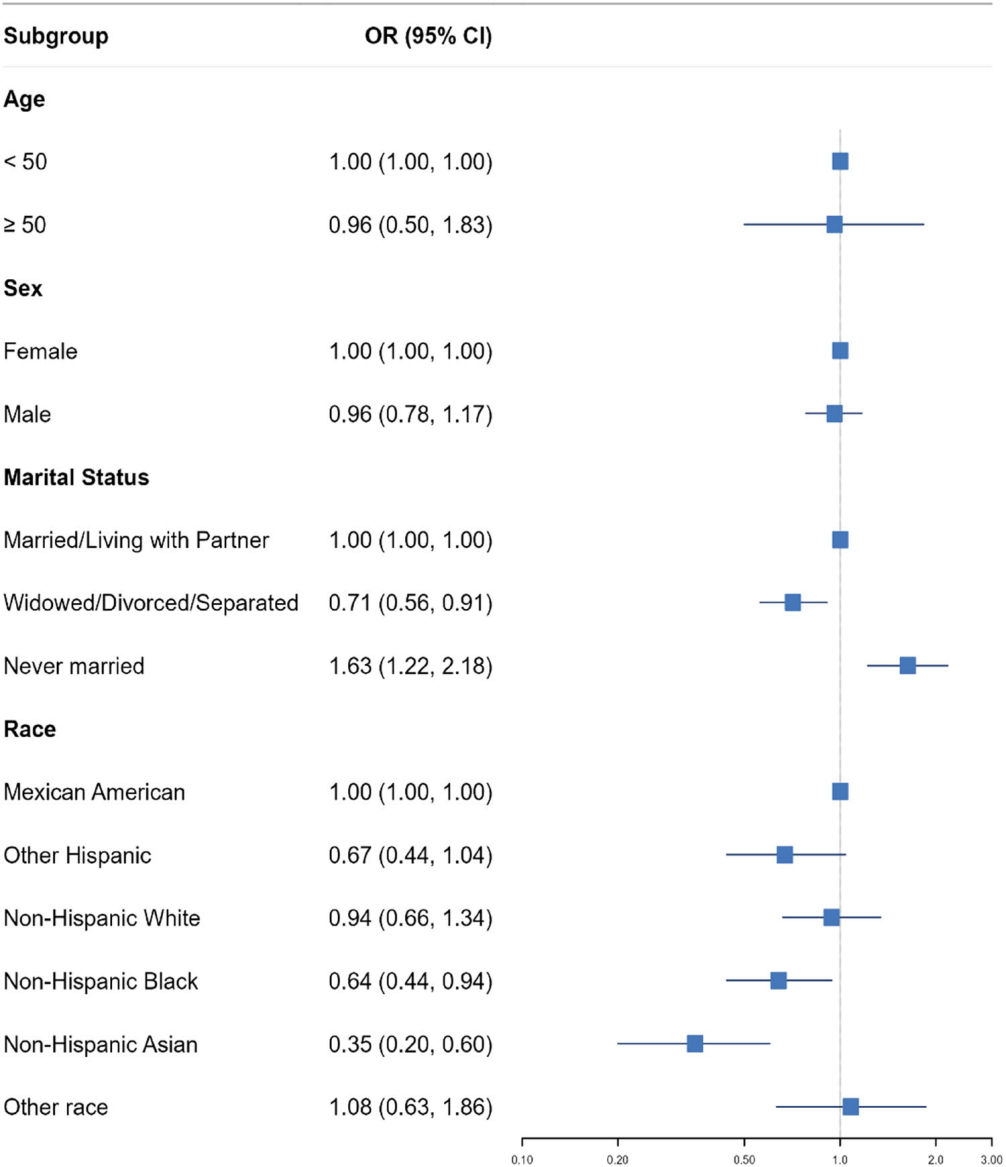
Subgroup analysis illustrating the link between dietary antioxidant quality score and the odds of developing epilepsy. Results are presented after adjusting for potential confounders in the fourth model.

As illustrated in Figure 2, an extensive subgroup analysis was executed, taking into account a multitude of variables, encompassing gender, age, education, marital status, and race. Our findings revealed that the highest adherence to the DAQS in participants with refused education and never married status was significantly linked to a 5.6‐fold and 63% increase in the odds of epilepsy compared to those with less than a nineth grade education (OR: 5.6, 95% CI: 1.00–10.06, *p* = 0.05) and married status (OR: 1.63, 95% CI: 1.22–2.18, *p* = 0.001), respectively. Although, a 43%, 67%, 82%, and 65% significant decrease in epilepsy odds was observed for individuals with 9th–11th grade (OR: 0.57, 95% CI: 0.36–0.88, *p* = 0.013), high school graduate (OR: 0.33, 95% CI: 0.22–0.50, *p *< 0.001), some college (OR: 0.18, 95% CI: 0.12–0.28, *p* < 0.001), and college graduate education (OR: 0.35, 95% CI: 0.22–0.53, *p* < 0.001) compared to those with less than a nineth grade education. Furthermore, widowed participants exhibited a 29% meaningful reduction in the odds of epilepsy compared to those with married status (OR: 0.71, 95% CI: 0.56–0.91, *p* = 0.006). Additionally, non‐Hispanic Black and Asian participants displayed a 36% and 65% diminished odds of epilepsy compared with Mexican American participants (OR: 0.64, 95% CI: 0.44–0.94, *p* = 0.024; OR: 0.35, 95% CI: 0.20–0.60, *p *< 0.001, respectively).

## 4 Discussion

Our analysis of NHANES data revealed significant differences in baseline characteristics between individuals with and without epilepsy, including age, education level, BMI, waist and hip circumferences, and various biochemical and dietary indicators. These differences are clinically relevant, as they not only underscore demographic and metabolic distinctions between groups but may also reflect underlying pathophysiological processes associated with epilepsy. For example, the lower BMI and reduced waist and hip circumferences observed among epilepsy cases may indicate altered energy metabolism or medication‐related effects. Additionally, higher HDL levels and lower triglycerides suggest favorable lipid profiles in cases, potentially reflecting dietary or metabolic adaptations (Kim and Cho 2019). The reduced ferritin and TIBC levels may indicate disturbances in iron homeostasis, which could exacerbate oxidative stress—a mechanism strongly implicated in epileptogenesis (N. Yang et al. 2020).

Nutritionally, individuals with epilepsy reported higher intake of carbohydrates and several antioxidants, including vitamins A, B6, B12, folic acid, calcium, and potassium, while consuming lower levels of zinc and selenium. These intake patterns may reflect conscious or unconscious dietary modifications due to epilepsy management, although reverse causation cannot be ruled out.

Importantly, after adjusting for potential confounders (age, sex, education, and race), a statistically significant inverse association was identified between DAQS and epilepsy odds in Model 4 (OR: 0.76; 95% CI: 0.68–0.85). While this association was not significant in earlier models, the findings in the fully adjusted model suggest that sociodemographic variables mediate or confound the relationship between dietary antioxidant quality and epilepsy. These variables—particularly education level and marital status—are known to influence dietary behavior, access to health resources, and health outcomes more broadly (Gherasim et al. 2020). For instance, lower education is linked to both suboptimal diet quality and higher epilepsy risk (Thowfeek et al. 2023), thereby strengthening the rationale for including these covariates in explanatory models.

Our findings should be interpreted with caution, as the observational nature of the study precludes causal inferences. Nonetheless, the effect size, though statistically significant, is modest. This raises questions about the clinical relevance of the association. We emphasize that our findings are hypothesis‐generating and should not be interpreted as evidence of causality due to the observational nature of the study.

Our subgroup analyses revealed variability in the association between DAQS and epilepsy across demographic strata. For instance, never‐married individuals had significantly higher epilepsy odds, while widowed participants and non‐Hispanic Black and Asian individuals showed lower odds. However, some subgroup estimates, such as the refused education category, exhibited wide CIs and limited precision, suggesting instability in the effect estimates. These findings should be interpreted with caution and considered exploratory in nature rather than definitive. The small sample sizes in certain strata may lead to overestimation or underestimation of effects and limit generalizability. These findings should be interpreted with caution, but they suggest possible effect modification by social determinants. Mechanistically, psychosocial stressors associated with marital status or limited educational attainment may influence oxidative stress levels via dysregulation of the hypothalamic‐pituitary‐adrenal axis, thereby contributing to increased neuronal excitability and seizure susceptibility (Maguire and Salpekar 2013). These findings align with a broader body of literature highlighting how structural social factors influence both diet quality and neurological outcomes (Solomou et al. 2023).

Our study is the first to evaluate the relationship between DAQS and epilepsy. In contrast, previous research has largely focused on the CDAI or individual antioxidant components. For instance, Y. Zhang et al. (2024) found an inverse association between CDAI and epilepsy, particularly in the higher quartiles (Q3 and Q4), and reported negative associations with vitamin A and zinc intake (Y. Zhang et al. 2024). These findings support the idea that dietary antioxidants may modulate seizure risk. Compared to CDAI, DAQS provides a user‐friendly and population‐independent approach, which may enhance clinical utility and generalizability. Furthermore, it directly aligns with dietary guidelines, making it a potentially more actionable index for public health and nutrition professionals.

Mitochondrial oxidative stress has been identified as a key contributor to epileptogenesis. This process involves excessive generation of ROS and imbalance in ROS/reactive nitrogen species (RNS) homeostasis, leading to oxidative damage of lipids, DNA, and proteins and resulting in neuronal hyperexcitability (Rafati et al. 2023; Lim and Thomas 2020, N. Yang et al. 2020). The brain's high oxygen demand and limited intrinsic antioxidant defenses make it particularly vulnerable. Antioxidants such as GSH, vitamin E, SOD, and catalase play critical roles in counteracting oxidative stress but can become depleted in epilepsy (Chidambaram et al. 2024; Liang et al. 2024; Yilgor and Demir 2024, X. Zhang et al. 2022). Animal models and human studies support these findings, demonstrating improved seizure outcomes with antioxidant supplementation. Clinical and preclinical data support these mechanisms. A pilot study in drug‐resistant epilepsy patients found that the ketogenic diet (KD) increased brain GSH levels up to sevenfold in gray matter and 14‐fold in white matter, correlating with substantial reductions in seizure frequency and duration (Hazany et al. 2023). Similarly, antioxidant therapy combining NADPH oxidase inhibition and Nrf2 pathway activation reduced ROS production and neuronal damage in seizure models (Shekh‐Ahmad et al. 2019). These studies collectively support the biological plausibility of dietary antioxidants modulating seizure susceptibility.

However, prior findings on individual antioxidants remain mixed. El‐Rashidy et al. (2020) reported lower selenium levels among epileptic patients, particularly those on KD. On the other hand, a Mendelian randomization study by Huang et al. (2024) found genetically predicted higher zinc levels associated with increased epilepsy risk—contrasting with our findings. This discrepancy may reflect several factors: the long‐term systemic exposure measured via genetic proxies versus short‐term dietary intake; a possible U‐shaped dose‐response relationship; or differences in zinc bioavailability and interaction with other nutrients (N.‐N.Chen et al. 2019). Animal models also suggest complexity: zinc deficiency has been shown to exacerbate hippocampal injury and cognitive dysfunction following seizures, while zinc supplementation improved outcomes (N.‐N.Chen et al. 2019). In a separate rodent study, a high‐fat diet increased seizure susceptibility, which was ameliorated by vitamin E supplementation through oxidative stress modulation (Alzoubi et al. 2019). These divergent findings underscore a key advantage of DAQS, which captures the synergistic effects of multiple antioxidants rather than focusing on isolated nutrients. This holistic approach may be more reflective of real‐world dietary patterns and biological complexity.

### 4.1 Strengths and Limitations

Our study benefits from several methodological strengths. NHANES employs a stratified multistage sampling design, yielding nationally representative estimates. The use of two 24‐h dietary recalls, combined with supplement data, enhances the accuracy of nutrient intake estimates. Multivariable models and quantile‐based DAQS analyses allowed for nuanced examination of associations while controlling for potential confounders including age, gender, race/ethnicity, education, and marital status.

Nonetheless, several limitations must be acknowledged. First, the cross‐sectional design of NHANES precludes causal inference and limits our ability to determine the temporal sequence between antioxidant intake and epilepsy development. Second, epilepsy case identification was based on recent prescription medication use, which may underestimate prevalence and result in misclassification. This approach may exclude untreated or undiagnosed individuals and include patients with alternative seizure etiologies or off‐label medication use. Third, although we adjusted for key sociodemographic and clinical covariates, residual confounding from unmeasured factors such as physical activity, environmental exposures, or genetic predisposition remains possible. Fourth, the DAQS includes only five dietary antioxidants and does not capture other potentially neuroprotective components like omega‐3 fatty acids, flavonoids, or dietary fiber (Kim and Cho 2019; Parsons et al. 2022). Lastly, the reliance on 24‐h recall may not fully capture habitual intake, and findings may differ with more comprehensive dietary assessment tools. These limitations highlight directions for future research, including refinement of antioxidant scoring systems. Longitudinal cohort studies with repeated dietary assessments preceding epilepsy onset are needed to establish temporality and examine causality. Mechanistic studies exploring how specific nutrient combinations interact with oxidative pathways could clarify the biological underpinnings. Expanding DAQS or developing new indices that incorporate a wider array of neuroprotective nutrients may provide a more complete picture of diet–epilepsy interactions.

### 4.2 Clinical and Public Health Implications

While further research is needed, our findings suggest that dietary antioxidant quality—as captured by DAQS—may be a modifiable risk factor for epilepsy. Clinically, DAQS could be used to identify individuals at higher risk based on dietary patterns and to guide dietary counseling. Public health initiatives aiming to improve diet quality—particularly among disadvantaged groups where stronger associations were observed—may yield broader neurological benefits. Given the safety and accessibility of antioxidant‐rich foods (e.g., fruits, vegetables, nuts), even modest benefits could translate to meaningful reductions in epilepsy burden at the population level. It is important to note that due to the cross‐sectional design of our study, causality cannot be established. Therefore, our results should be interpreted as associative and hypothesis‐generating, requiring validation in prospective cohorts or intervention trials.

## 5 Conclusion

In this cross‐sectional study, we observed a modest but statistically significant inverse association between higher DAQS and the odds of epilepsy. This association was most evident in fully adjusted models, suggesting that sociodemographic factors such as education level, marital status, and race/ethnicity may influence the relationship between dietary antioxidant intake and epilepsy risk. Specifically, individuals with a high school education or higher, as well as widowed participants and those identifying as non‐Hispanic Black or Asian, were found to have lower odds of epilepsy. These findings suggest a potential role for dietary antioxidants in epilepsy risk reduction, pending confirmation by longitudinal studies. Expanding dietary assessment tools to include additional nutrients and exploring how cultural, geographic, and social determinants interact with dietary intake will also be essential for advancing evidence‐based dietary recommendations in epilepsy prevention and care. These findings provide preliminary evidence that dietary antioxidants may play a role in epilepsy risk reduction; however, given the cross‐sectional design's inability to establish causality or evaluate long‐term effects, clinical applications remain speculative and require validation through longitudinal studies.

## Author Contributions

This study was conceptualized and designed by H.A. The primary manuscript was collaboratively authored by H.A., S.K., and M.M.A. H.A. executed data preprocessing and analytical procedures and compiled all tables and figures. The manuscript underwent review and editing by G.E., who additionally provided oversight throughout the process. All authors have thoroughly reviewed and granted their approval for the final iteration of this manuscript.

## Funding

The authors have nothing to report.

## Ethics Statement

The participants involved in this study were reviewed and approved by National Center for Health Statistics Research Ethics Review Board. All procedures in this study were carried out in compliance with relevant guidelines/regulations and the Declaration of Helsinki. The patients/participants provided their written informed consent to participate in this study.

## Conflicts of Interest

The authors declare no conflicts of interest.

## Peer Review

The peer review history for this article is available at https://publons.com/publon/10.1002/brb3.71018.

## Supporting information




**Supplementary Table**: brb371018‐sup‐0001‐Tables.docx

## Data Availability

The datasets analyzed during the current study are available at the NHANES website https://www.cdc.gov/nchs/nhanes/.
