# Correction to “Dietary Antioxidant Quality Score and Epilepsy Odds in the US Adults: a Cross‐Sectional NHANES Study”

**DOI:** 10.1002/brb3.71103

**Published:** 2025-12-02

Abbasi H, Khoshdooz S, Abbasi MM, Eslamian G. Dietary Antioxidant Quality Score and Epilepsy Odds in the US Adults: A Cross‐Sectional NHANES Study. Brain and Behavior. 2025 Nov;15(11):e71018.

In the originally published version, the affiliation superscripts for the authors were incorrect. The superscript for “Hamid Abbasi” and “Mohammad Mehdi Abbasi” should be 1, and the superscript for “Sara Khoshdooz” should be 2. The correct order of affiliations, based on the current author order, is as follows:

Student Research Committee, Faculty of Nutrition and Food Technology, Shahid Beheshti University of Medical Sciences, Tehran, Iran

Faculty of Medicine, Guilan University of Medical Science, Rasht, Iran

Department of Cellular and Molecular Nutrition, Faculty of Nutrition and Food Technology, National Nutrition and Food Technology Research Institute, Shahid Beheshti University of Medical Sciences, Tehran, Iran

Second, two rows were missing from Table 4 in the original article. A corrected version of Table 4 has been provided.

**TABLE 4:** Odds ratios (ORs) and 95% confidence intervals (95% CIs) for the odds of epilepsy stratified by quantiles of energy‐adjusted dietary antioxidant quality scores.

DAQS	Energy‐adjusted dietary antioxidant quality scores							
	Model 1$OR (95% CI)	*P*‐value	Model 2$OR (95% CI)	*P*‐value	Model 3$OR (95% CI)	*P*‐value	Model 4$OR (95% CI)	*P*‐value
Continuous	1.01 (0.88, 1.16)	0.899	1.00 (0.87, 1.15)	0.983	1.00 (0.87, 1.16)	0.994	1.01 (0.87, 1.17)	0.896
Q1	Reference		Reference		Reference		Reference	
Q2	1.01 (0.78, 1.31)	0.944	1.01 (0.77,1.33)	0.950	1.00 (0.75,1.32)	0.867	0.99 (0.75,1.32)	0.954
Q3	0.86 (0.66, 1.13)	0.287	0.86 (0.65, 1.13)	0.272	0.85 (0.64, 1.13)	0.322	0.86 (0.64, 1.15)	0.300
Q4	0.93 (0.71, 1.23)	0.629	0.91 (0.68, 1.21)	0.526	0.91 (0.67, 1.22)	0.558	0.90 (0.66, 1.21)	0.476
Q5	0.83 (0.64, 1.09)	0.188	0.80 (0.60, 1.06)	0.114	0.78 (0.58, 1.04)	0.147	0.76 (0.68, 0.85)	<0.001
*P _trend_*	0.831		0.752		0.780		0.076	

Abbreviation: OR: odds ratio; CI: confidence interval

* Binary logistic regression analysis was employed to derive ORs and their corresponding 95% CIs. The overarching trend of the OR across ascending quantiles was investigated by regarding the median score within each quantile as a continuous predictor variable.

Model 1: crude model

Model 2: adjusted for gender and age

Model 3: additional adjustments were applied for education level

Model 4: additional adjustments were applied for race and marital status

The authors declare that these amendments do not change the results or conclusions of the paper and apologize for any inconvenience caused by these oversights.

