# Supplementary material for: Dietary Antioxidant Quality Score and Epilepsy Odds in the US Adults: A Cross‐Sectional NHANES Study
Source: Brain Behav. 2025 Oct 29;15(11):e71018. doi: 10.1002/brb3.71018 (PMC12572107; doi:10.1002/brb3.71018)
Supplement: Supplementary file 1 — Supplementary Table: brb371018‐sup‐0001‐Tables.docx [file BRB3-15-e71018-s001.docx]

**Supplementary Table 1.** Other Biochemical and Anthropometric Characteristics of Study Participants by Epilepsy Status and DAQS Quantiles

|  | **Epilepsy** | | **Energy-adjusted dietary antioxidant quality score** | | | | | ***P*-value^2^** |
| --- | --- | --- | --- | --- | --- | --- | --- | --- |
|  | **Yes**  **n = 1086^1^** | **No**  **n = 1086^1^** | **Q1**  **n = 424^1^** | **Q2**  **n = 416^1^** | **Q3**  **n = 370^1^** | **Q4**  **n = 423^1^** | **Q5**  **n = 461^1^** |  |
| **Body Mass Index (kg/m^2^)** | 28.85 ± 6.52 | 31.46 ± 7.84 | 30.37 ± 6.81 | 30.09 ± 7.49 | 29.88 ± 7.28 | 30.16 ± 7.46 | 30.18 ± 7.78 | <0.0001 |
| **Waist circumference (cm)** | 100.39 ± 18.17 | 105.98 ± 16.85 | 103.35 ± 16.93 | 102.95 ± 18.12 | 102.36 ± 16.86 | 103.12 ± 17.58 | 103.82 ± 19.09 | <0.0001 |
| **Hip circumference (cm)** | 104.99 ± 14.06 | 110.21 ± 15.19 | 107.74 ± 13.87 | 107.52 ± 15.38 | 107.65 ± 14.98 | 107.75 ± 15.32 | 107.58 ± 15.27 | <0.0001 |
| **LDL-c (mg/dl)** | 97.64 ± 32.19 | 98.38 ± 37.19 | 96.68 ± 40.01 | 98.47 ± 33.14 | 104.28 ± 30.47 | 97.91 ± 32.45 | 94.70 ± 36.74 | 0.736 |
| **Triglycerides (mg/dl)** | 104.88 ± 58.53 | 125.65 ± 100.58 | 110.13 ± 67.88 | 111.73 ± 63.52 | 111.75 ± 67.16 | 115.85 ± 122.31 | 118.28 ± 73.01 | <0.0001 |
| **Total cholesterol (mg/dl)** | 185.41 ± 40.98 | 179.54 ± 43.06 | 180.11 ± 42.66 | 182.99 ± 42.22 | 189.59 ± 38.48 | 183.20 ± 43.80 | 178.39 ± 42.54 | <0.0001 |

Abbreviations: LDL-c: low-density lipoprotein-cholesterol; Data are presented as Mean ± SD

1 Individuals in the first quantile of DAQS had DAQS score less than (0.004); second quantile: between (0.004) and (0.04); third quantile: between (0.04) and (0.17); fourth quantile: between (0.17) and (0.47) and fifth quantile: more than 0.47.

2 Derived from independent Student's t-test.
